# Application of the NucliSENS easyMAG system for nucleic acid extraction: optimization of DNA extraction for molecular diagnosis of parasitic and fungal diseases

**DOI:** 10.1051/parasite/2013051

**Published:** 2013-12-12

**Authors:** Fakhri Jeddi, Renaud Piarroux, Charles Mary

**Affiliations:** 1 Aix-Marseille Université, Faculté de Médecine, UMR MD3 13284 Marseille France; 2 APHM, Hôpital de la Timone, Laboratoire de Parasitologie-Mycologie 13385 Marseille France

**Keywords:** Nucleic acids extraction, Molecular diagnosis, Parasitic diseases, qPCR

## Abstract

During the last 20 years, molecular biology techniques have propelled the diagnosis of parasitic diseases into a new era, as regards assay speed, sensitivity, and parasite characterization. However, DNA extraction remains a critical step and should be adapted for diagnostic and epidemiological studies. The aim of this report was to document the constraints associated with DNA extraction for the diagnosis of parasitic diseases and illustrate the adaptation of an automated extraction system, NucliSENS easyMAG, to these constraints, with a critical analysis of system performance. Proteinase K digestion of samples is unnecessary with the exception of solid tissue preparation. Mechanically grinding samples prior to cell lysis enhances the DNA extraction rate of fungal cells. The effect of host-derived nucleic acids on the extraction efficiency of parasite DNA varies with sample host cell density. The optimal cell number for precise parasite quantification ranges from 10 to 100,000 cells. Using the NucliSENS easyMAG technique, the co-extraction of inhibitors is reduced, with an exception for whole blood, which requires supplementary extraction steps to eliminate inhibitors.

## Abstract (fr)

Durant les vingt dernières années, l’avènement des techniques de biologie moléculaire a révolutionné le diagnostic des maladies parasitaires sur le plan de la rapidité, de la sensibilité et du typage des parasites. Cependant l’extraction des acides nucléiques reste une étape critique qui doit être adaptée au diagnostic et à l’épidémiologie. L’objectif de ce travail est d’illustrer les contraintes liées à l’extraction d’ADN en vue du diagnostic des parasitoses et de proposer les adaptations nécessaires à l’utilisation d’un système automatisé, NucliSENS easyMAG, en procédant à une analyse critique de ses performances. Ce système permet d’éviter le prétraitement par la protéinase K à l’exception des tissus solides. Le broyage mécanique avant la lyse cellulaire améliore l’extraction de l’ADN des cellules fongiques. L’effet des acides nucléiques dérivés de l’hôte sur le rendement d’extraction de l’ADN du parasite varie en fonction de la densité en cellules de l’échantillon de l’hôte. Le nombre optimal de cellules pour une quantification précise des parasites varie de 10 à 100,000 cellules. En utilisant la technique NucliSENS easyMAG, la co-extraction des inhibiteurs est réduite, avec une exception pour le sang total, qui nécessite des étapes d’extraction supplémentaires pour éliminer les inhibiteurs.

## 1. Introduction

The field of molecular diagnosis and epidemiology of parasitic diseases and mycoses is constantly evolving. As is the case for other infectious diseases, diagnostic efforts have primarily focused on developing polymerase chain reaction (PCR) assays for a wide spectrum of parasitic diseases. Based on a Medline literature review (January 2013) of terms matching “PCR diagnosis of parasitic diseases” in reports published during the last 22 years, seven references were found for the year 1990, 99 for 1995, 170 for 2000, 329 for 2005, 522 were found for 2010, and 637 for 2012. The total number of references for the period 1990–2012 was 5,398. Most PCR assays are employed for disease diagnosis, epidemiologic studies, or polymorphism-based isolate genotyping [13, 17]. Unlike the evolution of PCR technology, optimization of DNA extraction techniques has remained neglected. In comparison to PCR-based diagnoses, the term “nucleic acids extraction and parasitic diseases” was referenced in Medline in 274 articles published between 1990 and 2012. Current extraction methods are based on silica adsorption, which are more convenient and safer than the former phenol chloroform methods. Routine laboratory analysis led to an automation of the process and highlighted the need to adapt the technique based on the characteristics of the biological samples.

Currently, automated nucleic acid extraction can be performed using two approaches as follows:

- mechanical handling of the silica columns, which is complex due to the washing steps that require either centrifugation or vacuum.
- application of silica coupled to magnetic particles, which is more efficient and can be automated. At least five commercially available systems incorporate this methodology (QiagenEZ1 and QIAsymphony, both from Qiagen; the Maxwell 16 System from Promega BioSciences; the MagNA Pure system from Roche Diagnostics and the NucliSENS easyMAG system from BioMerieux). The principal difference between these systems involves the nature of the lysis process. The NucliSENS easyMAG system employs strong denaturing reagents, while the other techniques involve a milder lysis step using tensio-active agents associated with enzymatic proteolysis of the sample. However, the NucliSENS easyMAG process must be optimized for the molecular diagnosis of parasitic diseases and mycosis, as the operating protocols were initially established for the molecular diagnosis of viral diseases.

The aim of this study was to adopt sample processing and optimize the method for parasitic load quantification using the semi-automated NucliSENS easyMAG system for nucleic acid extraction. In this report, we assess system performance, limits, and specific requirements for parasitological studies.

## 2. Materials and methods

### 2.1 Study design

**Figure 1.**
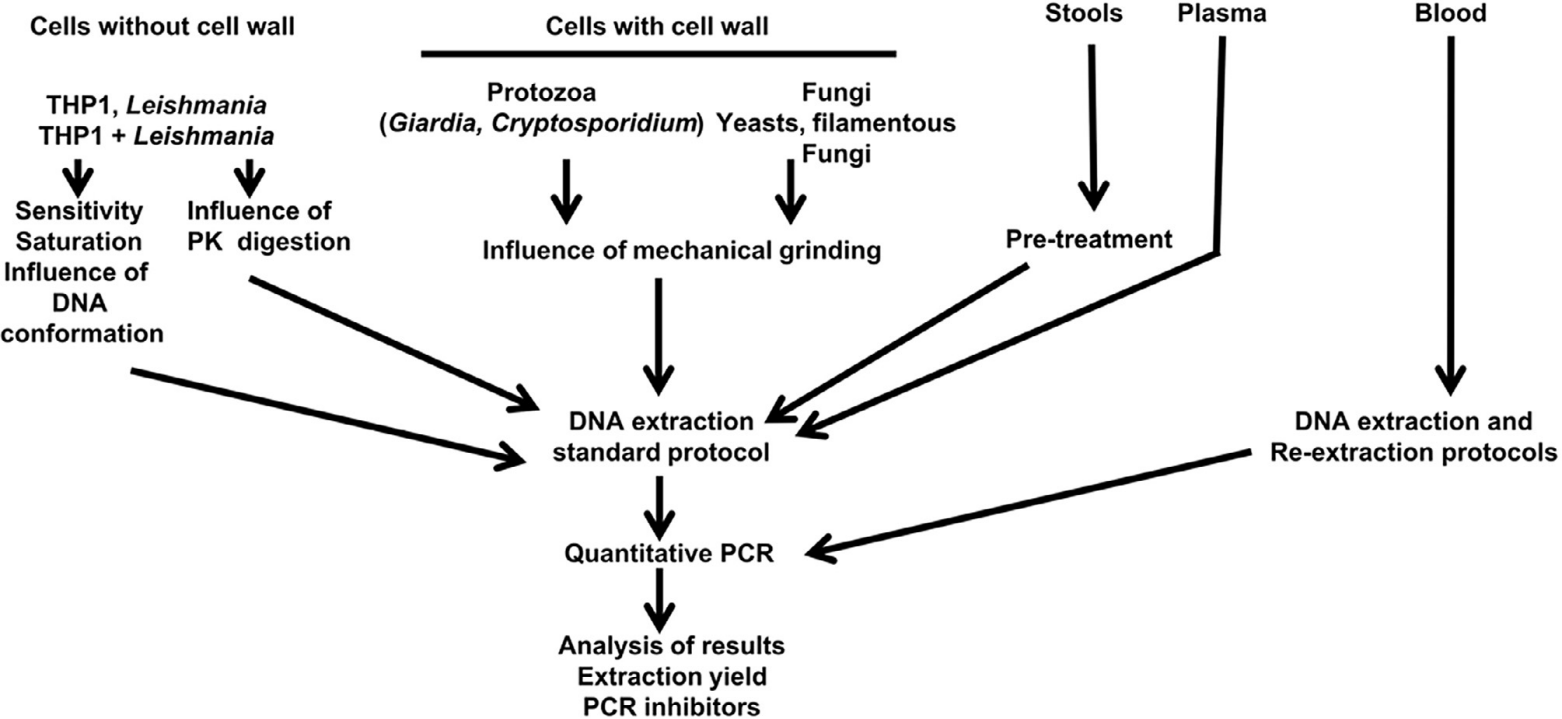
Study design.

Our goal was to optimize sample processing variables such as the optimal cell number range for parasite quantification, sample pre-treatment with proteinase K (PK) digestion, mechanical grinding, and the detection of PCR inhibitors in the recovered DNA solution. The experiments were assessed via quantitative PCR (qPCR). These elements are summarized in Figure 1.

### 2.2 Nature of the samples

We analyzed a panel of various types of cells and tissues that are commonly encountered during the routine diagnosis of parasitic diseases.

The THP1 cell line (ATCC TIB-202), which was grown in RPMI medium (Gibco ref. 52400-025, Life Technologies), represented the host cells and was used to analyze the sensitivity and saturation of the extraction process.

*Leishmania infantum* (MCAN/82/GR/MON497) promastigotes, which were representative of protozoa without cell wall or cystic stages, were grown in RPMI medium. This parasite harbors two kinds of nucleic acids: the nuclear DNA and the kinetoplastic DNA, essentially composed of small circular supercoiled double-stranded DNA (minicircles). This property will allow us to study the possible difference of affinity of these molecules for the silica by performing extractions on various numbers of cells and simultaneous quantification of nuclear and kinetoplastic targets.

To test the influence of system saturation with human DNA, either artificial samples were prepared by mixing THP1 cells with *Leishmania* at various proportions or the cells were tested separately. PCR inhibition by residual hemoglobin was assessed following DNA extraction of human blood mixed with *Leishmania* parasites.

Stool samples containing either *Cryptosporidium* oocysts (60 positive samples out of 130 samples as assessed via microscopic examination) or *Giardia* cysts (four samples) represented the cystic stage of protozoa. Diagnosis was established via microscopy. As stool samples represent a complex medium, 70 samples without parasitic elements were also included to assess for the removal of inhibitors.

*Candida* and *Aspergillus* harbor a cell wall that protects from cell lysis. We used *Candida albicans* cells (ATCC 10231) and *Aspergillus fumigatus* mycelium (ATCC 13073), which were grown in Sabouraud’s liquid medium for 4 days.

Assays were performed on 820 plasma samples and 428 bronchoalveolar lavages (BAL) for the detection of *Aspergillus* DNA, of which 18 were positive. All human-derived samples were anonymized according to the French legislation on Biological Research.

### 2.3 Mechanical grinding of samples

As encountered with plant DNA purification [10], mechanical disruption improves the yield of DNA extraction from cells with a cell wall or parasite cysts, prior to chemical and/or enzymatic lysis.

We tested two mechanical grinding devices as follows:

- a vortex (Vortex-Genie 2, Scientific Industries) with a tube holder (MO BIO vortex adapter ref. 800-606-6246, MO BIO Laboratories) and 2-mL tubes containing approximately 25 glass beads (Sigma ref. G1152).
- a high-power mechanical grinder (FastPrep 24, MP Biomedicals) set at maximum power for 1 min, using disposable tubes containing ceramic beads (Lysing Matrix D, MP Biomedicals).

In these conditions, heating does not exceed 35 °C so a cooling device was considered unnecessary.

### 2.4 Biological sample pre-treatment

#### 2.4.1 Stool samples

Stool samples (200 mg) were suspended in 800 μL of lysis buffer in a microtube containing ceramic beads. After a 1-min shaking step using the FastPrep system at max power and 10-min incubation at room temperature, the microtubes were centrifuged for 10 min at 10,000 g and 200 μL of supernatant was submitted to extraction.

#### 2.4.2 Blood samples

Whole blood (sample volumes less than 250 μL) was directly applied for extraction, as recommended by the manufacturer. For larger sample volumes, we tested re-extraction methods as described below.

#### 2.4.3 Cell suspensions

Cells lacking a cell wall were suspended directly in lysis buffer.

Cells protected by a cell wall, such as yeasts, filamentous fungi, and protozoan cysts, or host tissues likely to contain these elements were mechanically ground in lysis buffer prior to extraction.

#### 2.4.4 Plasma samples

Plasma, which was mixed directly with lysis buffer prior to extraction, was used to detect free *Aspergillus* DNA.

### 2.5 Proteinase K digestion

We analyzed the effects of PK digestion prior to DNA extraction from THP1 cells and *Leishmania*. All PK (Qiagen ref. 19131) treatments were performed overnight at 2 mg/mL (final concentration) in 0.1X ATL lysis buffer (Qiagen ref. 19076) at 56 °C.

### 2.6 NucliSENS easyMAG technology

NucliSENS easyMAG technology is based on the Boom technique [5], utilizes magnetic silica particles, and processes 24 samples simultaneously. This technology allows for the dynamic capture of nucleic acids and the washing of silica without loss of material.

#### 2.6.1 Sample lysis

Boom lysis buffer contains 5 M guanidinium thiocyanate, a powerful dissociating and denaturing agent, plus tensio-active compounds. Direct lysis is the most frequently used technique; the sample volume should not exceed 1 mL to respect the ratio between sample volume and lysis buffer volume (1 volume for 2 volumes, respectively). External lysis saves time and enhances the safety of sample manipulation and transport.

#### 2.6.2 DNA adsorption

Adsorption of nucleic acids occurs in lysis buffer at neutral pH levels. The volume of the magnetic silica suspension can vary between 50 μL and 140 μL (as recommended by the manufacturer). Preliminary experiments showed that 50 μL of silica is sufficient for samples with cell numbers inferior to 10^4^. However, 140 μL of silica suspension is required for high-density samples, such as blood and solid tissues. All assays described in the current report were performed using 140 μL of silica suspension. Silica was added manually and homogenized thoroughly.

#### 2.6.3 Washing

The initial washing step was performed using a buffer containing guanidinium thiocyanate to maintain high stringency, and the final steps were performed at a neutral pH level with high salt concentrations to retain the nucleic acids adsorbed on the silica matrix.

#### 2.6.4 Elution

The elution step was performed at 70 °C in a volume that can vary between 25 μL and 110 μL at pH >8.0. The choice of elution volume essentially depends on the expected DNA quantity, the possible presence of inhibitors, and the volume required for amplification. The elution volume was 50 μL with an exception for the stool samples (elution volume 100 μL). The nucleic acids were stored at −40 °C.

#### 2.6.5 Re-extraction

The re-extraction experiments were performed on blood sample volumes superior to 250 μL, as residual hemoglobin present in the corresponding eluates inhibits PCR amplification.

We prepared three sets of five blood samples; each tube contained 400 μL of blood and 10^4^ *Leishmania*. Two re-extraction experiments were performed. The eluted DNA isolated from the first set of samples was processed using new magnetic silica and a new cassette. In contrast, the eluate isolated from the second set of samples was mixed a second time with the magnetic silica in the cassette used for the initial extraction.

A single extraction was performed on the third set, which served as a control.

An additional control consisted in direct extraction of 10^4^ washed *Leishmania* promastigotes.

### 2.7 QIAamp DNA Mini kit

We compared the DNA yield obtained from cell suspensions using the NucliSENS easyMAG system and the QIAamp DNA Mini kit (Qiagen ref. 51034). The QIAamp DNA Mini kit was used according to the manufacturer’s specifications, with the exception of PK digestion, which was extended to 12 h to obtain the highest extraction yield [14].

### 2.8 Quantitative PCR assays

A comparison between the various extraction assays was performed via PCR quantification of the targets present in each DNA sample. The samples were compared by means of absolute quantification or relative comparisons were based on the difference in cycle threshold (Ct) values obtained from the analysis of amplification curves.

The following qPCR assays were employed to quantify either human or parasitic DNA:

- human albumin gene [12].
- *Leishmania* kinetoplastic DNA [14] and *Leishmania* DNA polymerase [15].
- *Candida albicans*- and *Aspergillus fumigatus*-specific PCR [20].
- *Cryptosporidium*-specific PCR [8].
- *Giardia*-specific PCR [2].

Each of these systems employed hydrolysis probes (Taqman) as reporter and quantitative results were calculated applying standard curves generated by using serial dilutions of plasmid DNA containing the target sequences. TA cloning of the corresponding amplicons was performed using a TOPO TA cloning kit (Invitrogen ref. K4520-01, Life Technologies). Plasmids were amplified, purified from bacteria, and quantified via spectrophotometry prior to use. The results were expressed as the number of target sequence copies or the number of parasitic cells when the gene copy number was known.

### 2.9 Identification of inhibitors

The identification of inhibitors was performed on all samples using an M13 plasmid containing foreign DNA (Applied Biosystems part # 360364, Life Technologies). This plasmid was added to DNA samples to yield 20 copies per reaction tube. A qPCR assay targeting this plasmid (using M13 universal primers and a Taqman probe specific for the DNA insert) was performed for which the PCR was positive, thereby indicating the absence of inhibitors.

In addition to this initial assay, all samples assessed using *Aspergillus* DNA-specific qPCR were tested at two concentrations: 1μL of the eluate and 1μL of a 1/20 dilution of the eluate. The expected difference in Ct values between the two concentrations is 4.33 in the absence of inhibitors. We consider a difference in Ct values inferior to 3.5 as partial inhibition.

### 2.10 Statistical analysis

Statistical analyses were conducted using Statview 5 software (SAS Institute, Cary, USA).

## 3. Results

### 3.1 Proteinase K digestion is unnecessary for the treatment of *Leishmania* and human cell suspensions

**Figure 2.**
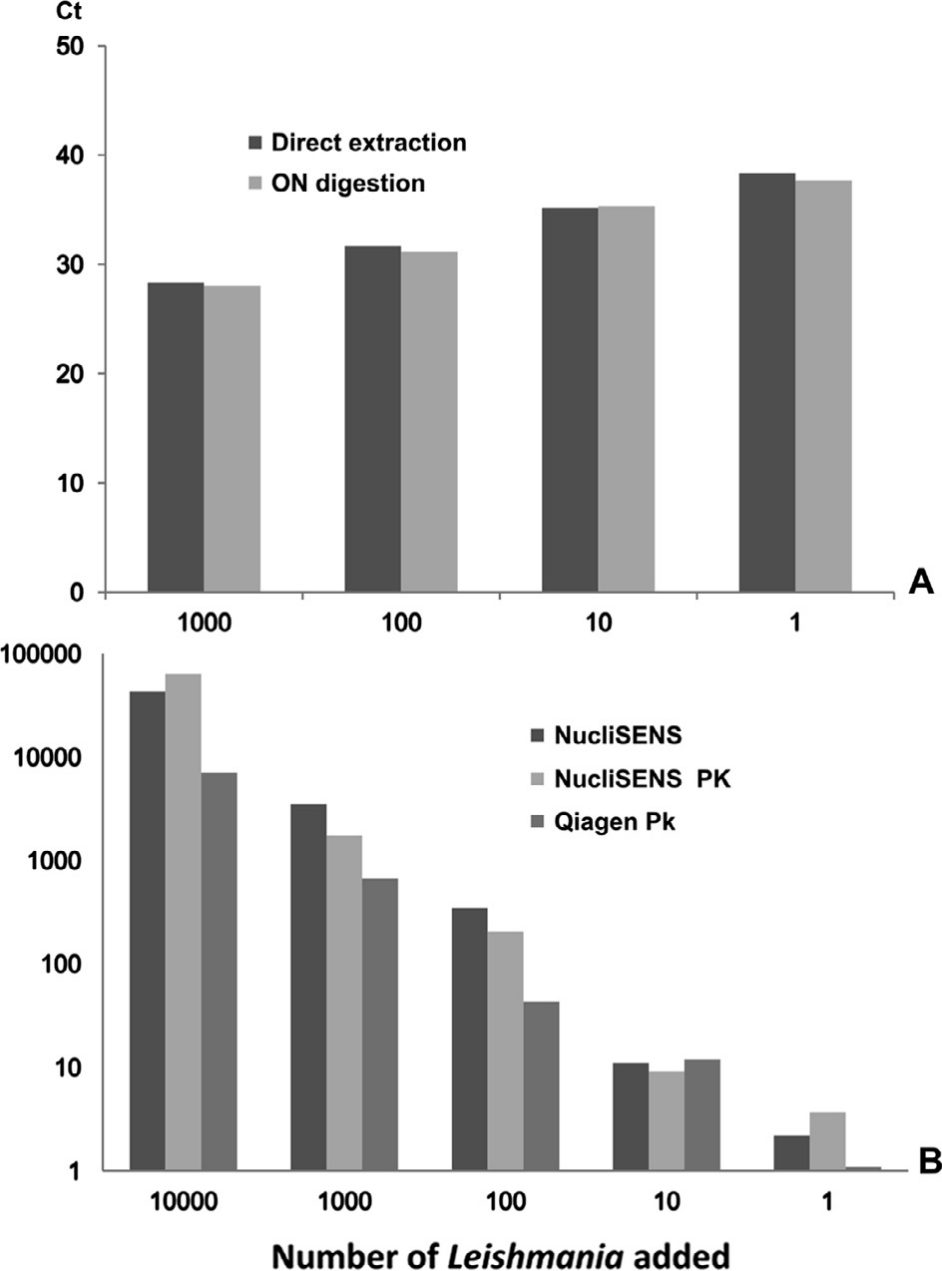
Influence of proteinase K digestion (56 °C overnight) on DNA extraction. Graph A shows the Ct values obtained by quantifying THP1 cell DNA derived from direct extraction with the NucliSENS easyMAG system and extraction performed on the same quantity of cells following overnight (ON) digestion with Proteinase K. Graph B shows *Leishmania* quantification after extraction with the NucliSENS easyMAG system both with and without PK and quantification after extraction using a QIAamp DNA Mini kit after ON digestion with PK.

Figure 2 presents the results of DNA extraction from THP1 cells (1A, ranging from 1 to 1,000) and *Leishmania* (1B, ranging from 1 to 10,000) and the influence of PK digestion. No significant differences were observed between the experiments performed with and without PK digestion. Extractions performed using a QIAamp DNA Mini kit following PK digestion of *Leishmania* showed comparable results.

### 3.2 Stability of the lysates

We prepared six identical lysates from stool samples containing *Cryptosporidium* and buffy coat samples containing *Leishmania*. Two of these lysates were extracted within 2 h after lysis, two were extracted after 3 days, and the remaining two were extracted after 1 week. The lysates were conserved at room temperature, mean Ct were 31.3 ± 0.25 and 18.85 ± 0.45 for *Cryptosporidium* and *Leishmania* respectively; we did not observe a significant variation between the results obtained after different storage times (*p* > 0.15).

### 3.3 Mechanically grinding cysts and fungi improves the extraction yield

We first assessed the influence of mechanical grinding on the DNA extraction yield with *Candida albicans* cells and *Aspergillus fumigatus* mycelium.

**Figure 3.**
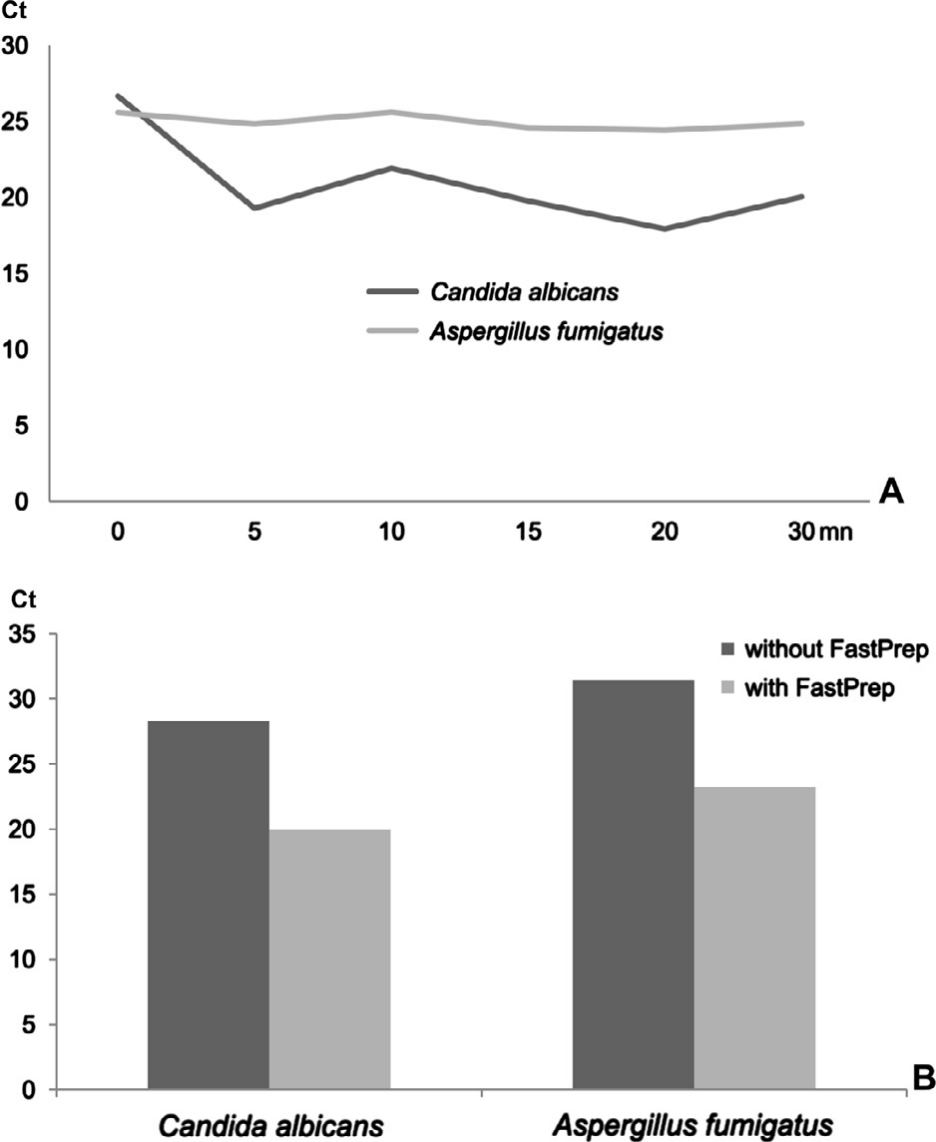
Results of the extraction experiments performed on yeast (*Candida albicans*) and filamentous fungi (*Aspergillus fumigatus*). A presents the kinetics of the extraction process after vortexing and glass-bead treatment. B shows the differences in DNA quantity obtained from fungal cells using the FastPrep system (with) compared to the same process without grinding.

Vortexing plus glass-bead treatment disrupted *Candida* cell wall after 5 min of agitation; however, this treatment was ineffective in disrupting the cell wall of filamentous fungi (Figure 3A).

The FastPrep system increased the extraction efficiency for both *Candida* and *Aspergillus*; the difference in Ct between direct lysis and lysis plus cell wall disruption was 7.8 and 8.8 for *Candida* and *Aspergillus*, respectively (Figure 3B). As the resulting values were multiplied by 222 (2^7.8^) and 445 (2^8.8^), respectively, the initial yield (without disruption) was estimated to be less than 0.5% of the initial fungal DNA content.

Mechanical disruption by means of the FastPrep instrument and ceramic beads was also performed on stool samples that were tested positive for *Cryptosporidium sp.* and *Giardia intestinalis* cysts. For each sample, equal quantities of material were subjected to extraction either before or after disruption. Table 1 presents the results of the corresponding qPCR assay. Each sample was tested in quadruplicate, and a 3.5-fold (*p* = 0.03) and a 66-fold (*p* = 0.005) increase was observed for *Cryptosporidium* and *Giardia*, respectively. Regarding the *Giardia* test, two out of four assays without mechanical disruption were negative (Table 1). Although these samples yielded negative PCR results, parasite cysts were observed by microscopy.

**Table 1.** Quantification of DNA following extraction from stool samples (*Cryptosporidium* and *Giardia*) and white blood cell samples (*Leishmania*). This table presents the number of positive experiments and the mean number of molecular targets detected from four different samples for each parasite. The same quantity of each sample was tested with and without FastPrep disruption.

	Without disruption		After FastPrep disruption		
	Positive PCR	Mean quantity Cells/tube	Positive PCR	Mean quantity Cells/tube	*p* value
*Cryptosporidium parvum*	4/4	547	4/4	1940	0.03
*Giardia intestinalis*	2/4	9.5	4/4	636	0.005
*Leishmania infantum*	4/4	309	4/4	285	NS

Regarding the *Leishmania* cell suspensions, mechanical disruption did not improve DNA yield.

### 3.4 The easyMAG system efficiently removed PCR inhibitors

**Figure 4.**
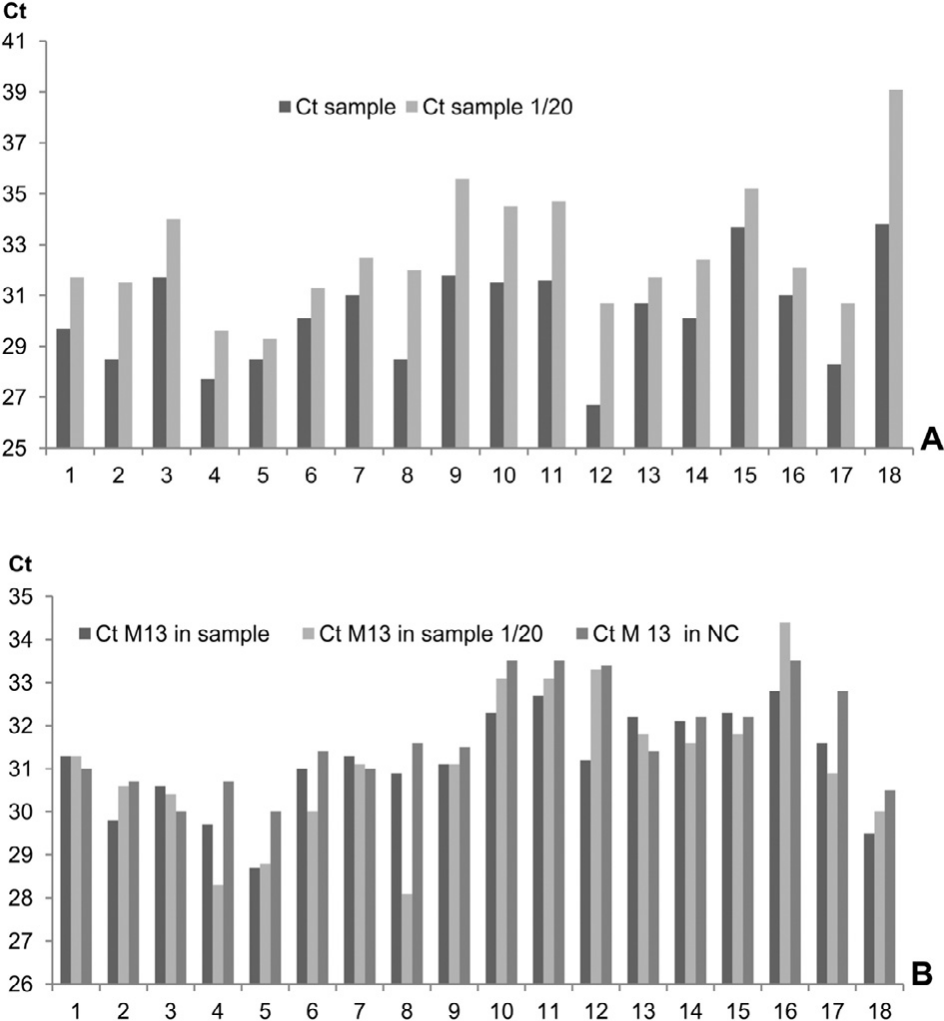
Identification of PCR inhibitors in 18 biological samples positive for *Aspergillus*. Graph A: Ct values obtained from pure and diluted DNA samples (dilution rate 1/20). Graph B: Ct values obtained with 20 copies of a plasmid DNA systematically added to the same biological samples (undiluted and diluted) and a negative control sample (NC).

We tested 1,280 samples (plasma and BAL) for the presence of *Aspergillus* DNA. We did not observe a situation in which both the 1/20 DNA dilution yielded a positive PCR result and the undiluted sample was negative. Furthermore, the M13 plasmid PCR was never inhibited. These results indicate the absence of total PCR inhibition. Eighteen samples were tested positive for *Aspergillus* DNA. Although, the plasmid PCR was not affected, we observed variations in Ct values between pure and diluted DNA, thereby suggesting the presence of inhibitors in some samples (Figure 4). In all cases, the residual inhibitor concentration was insufficient to induce complete inhibition of DNA amplification.

In DNA extracted from stool samples, 1 case of plasmid inhibition was observed among 130 samples.

The presence of residual hemoglobin after DNA extraction from whole blood is a known cause of PCR inhibition. Using the NucliSENS easyMAG system, PCR inhibition is frequently observed when more than 250 μL of blood is subjected to extraction. To avoid this problem, we tested three technical approaches, using 400 μL of blood containing 10^4^ *Leishmania*. We failed to amplify *Leishmania* DNA in eluate derived from a single extraction. In contrast, inhibitors were successfully removed following the re-extraction of DNA with either new silica matrix or the silica used for the initial extraction, and 10,880 and 8,600 parasites/tube were recovered, respectively. A single extraction performed directly on a suspension of 10^4^ purified *Leishmania* served as a control and yielded 10,400 parasites/tube.

### 3.5 Extraction yield varied with cell density

**Figure 5.**
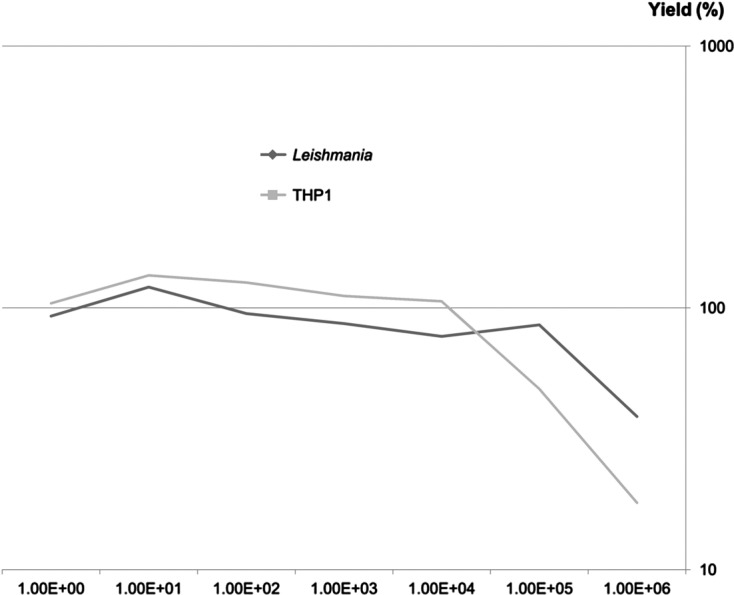
Yield of DNA extraction from *Leishmania* and THP1 cells using the NucliSENS easyMAG system.

We first tested the saturation limit of the silica matrix by testing increasing quantities of *Leishmania* or THP1 cells. Figure 5 presents the variation in DNA extraction yield in relation to cell number. For *Leishmania*, saturation of the silica matrix (140 μL is the largest volume recommended by the manufacturer) was observed with 10^5^ cells, while for THP1 monocytes, saturation was observed with greater than 10^4^ cells.

**Figure 6.**
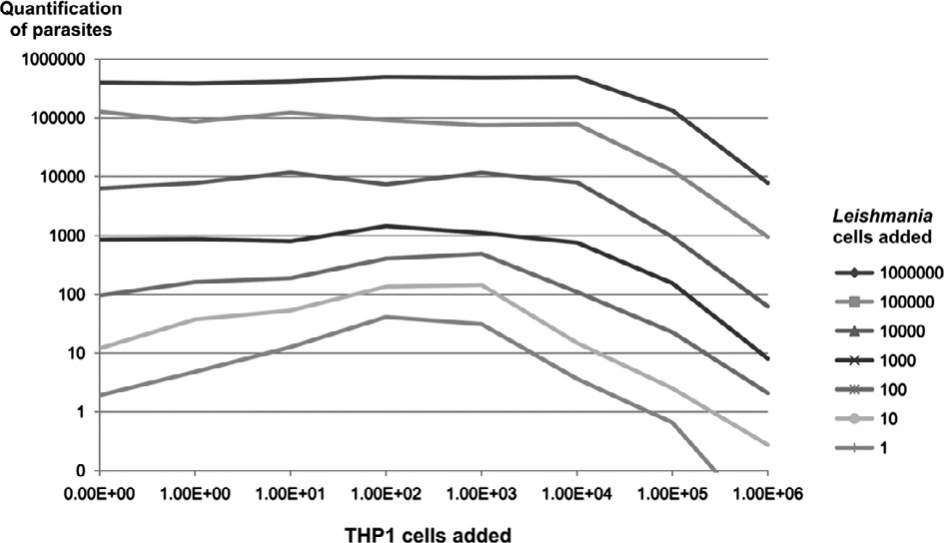
Influence of the quantity of human cells (THP1 cells) on *Leishmania* quantification at various concentrations of host cells and parasites.

Next, we performed an experiment to assess the influence of various quantities of host cells (THP1 ranging from 0 to 10^6^ cells) on the extraction of *Leishmania* DNA (ranging from 0 to 10^6^ cells). All DNA solutions were tested for *Leishmania* abundance in duplicate using a qPCR assay targeting kinetoplastic DNA. Composition of the samples and the results of *Leishmania* quantification are summarized in Figure 6. Parasitic DNA extraction was impaired when more than 10^4^ host cells were present in the sample, which is generally encountered in most human samples. Strikingly, the presence of 10^2^–10^3^ host cells seemed to enhance the extraction yield of low parasite-density samples.

**Figure 7.**
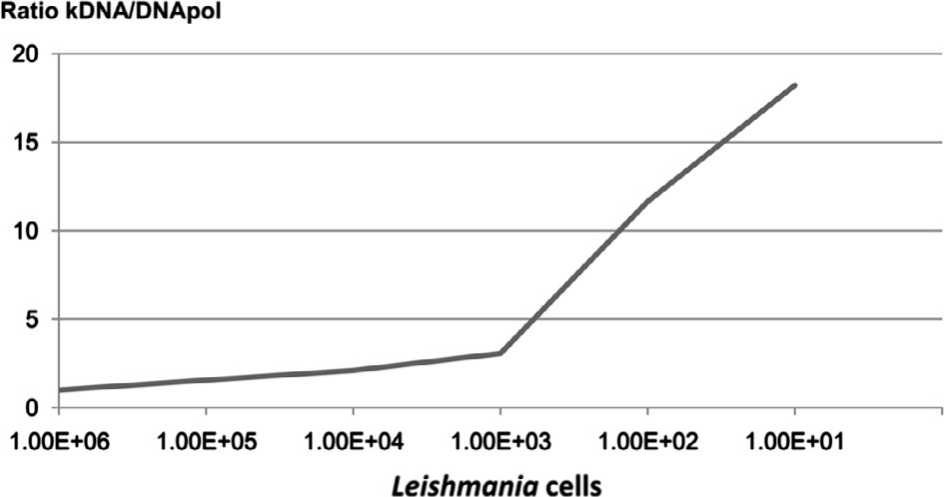
Variation of the ratio between kinetoplastic DNA and nuclear DNA extraction with various *Leishmania* quantities in the presence of 10^3^ THP1 cells.

To better assess this paradoxical effect, we tested the hypothesis of a differential affinity between double-stranded circular DNA (kinetoplastic DNA) and genomic DNA. We performed DNA extractions from *Leishmania* in the range of 10^2^–10^5^ in the presence of a constant number of human cells (10^3^). As shown in Figure 7, the affinity of the two types of parasitic DNA was nearly identical for high quantities of parasites (equal or superior to 10^3^), while for lower quantities, kinetoplastic DNA showed a higher affinity for the silica matrix.

## 4. Discussion

Nucleic acid extraction is the first step in the molecular diagnosis and molecular epidemiology of infectious diseases. Due to the low proportion of parasitic DNA relative to host nucleic acid present in samples, the extraction process should be optimized to achieve highly sensitive, parasite-specific sequence detection. For the molecular quantification of parasites, the DNA extraction step should also be quantitative.

Several factors influence the quality and quantity of extracted nucleic acids, including the nature and cell content of the biological samples, the presence of PCR inhibitors, the cell wall of bacteria or fungi, and the cystic stage of the parasite.

Regardless of the DNA isolation method, it is crucial to perform sample pre-treatment prior to nucleic acid purification for several reasons.

First, this step allows for cell concentration from large-volume or low-cell density samples. This concentration step may eliminate elements such as plasma, red blood cells, and anticoagulants, which contain molecules that may interfere with downstream assays. The presence of free parasitic DNA in biological fluids, particularly in circulating blood, has been described for aspergillosis [3, 24], candidiasis [11], and leishmaniasis [7]. This free parasite DNA can either be removed or retained during the extraction process depending on the aim of the study.

Second, protein denaturation and/or hydrolysis are necessary to obtain the optimal extraction yield. Most techniques involve PK digestion prior to cell lysis. In a previous study [14] using the QIAamp DNA Mini kit, we showed that the PK digestion step should be extended to obtain a near complete extraction, a key element for precise quantification. The degree of stringency of many lysis buffers included in commercial kits varies, and most kits involve mild denaturation steps with reagents that enable PK activity. Using the NucliSENS easyMAG system, PK digestion is unnecessary for cell suspensions, which saves time and prevents the loss of material. Strong dissociation is observed using the Boom technique with 5 M guanidinium thiocyanate [5], which inhibits PK digestion and other biological activities, even viral infectivity. However, PK digestion is necessary for the complete dissociation of solid tissues and should therefore be performed prior to the addition of such lysis buffer. In a recent study about molecular diagnosis of cryptosporidiosis [16], we tested nine extraction methods: two of them, Nucleospin and QiaAmp DNA, showed that proteinase K treatment allows cyst disruption as supplementary mechanical grinding does not improve the DNA output. Concerning *Giardia* cysts PK digestion seems to be insufficient for cyst disruption and mechanical breakage should be performed [1]. Third, the dissociation capacity of lysis buffer is generally insufficient to completely disrupt the cell wall of filamentous fungi or cystic stages of parasites. In these cases, the mechanical disruption of cells notably improves the extraction yield and gives better results than the enzymatic treatment with PK, thereby considerably enhancing the detection sensitivity of such pathogens as mentioned by Nawrot [18]. Although vortexing with glass beads was sufficient for yeast treatment, filamentous fungi extraction yield was only enhanced using high-power mechanical grinding. DNA extraction from filamentous fungi or yeast performed without mechanical disruption resulted in a low extraction yield, which may largely explain the lack of sensitivity observed in the molecular diagnosis of systemic mycoses [6] using conventional PCR or real-time PCR. Sonication has been described as the most powerful method for disrupting cell walls [9]; however, this process induces DNA fragmentation. Therefore, mechanical grinding remains an optimal alternative for DNA extraction.

A combination of powerful mechanical disruption and dissociation using Boom lysis buffer yielded a DNA output identical to that obtained using PK digestion and allowed for the recovery of nucleic acids in less than 1 h. The NucliSENS easyMAG system allows for extraction even from low-cell density samples, as this technique allows for the recovery of DNA from as few as 10 cells (as shown in Figures 2, 5, and 6). In this case, DNA samples should be recovered in a small elution volume to increase both the concentration and the probability of detecting pathogen DNA.

In contrast, the saturation limit of the easyMAG system is in the range of 10^4^–10^5^ cells, while some kits using silica columns are able to bind DNA from more than five million cells without a considerable loss [19]. Therefore, this technique requires a lower quantity of sample than the classical column extraction systems to achieve accurate quantification of both parasitic and host DNA [23]. Exceeding the saturation limit of the silica matrix would lead to underestimate the parasitic load of the biological sample [21] and to overestimate the real DNA content when preparing DNA standard solutions from quantified parasite’s suspensions. For qualitative analysis and epidemiological studies, the extraction yield does not affect the results (e.g., the presence or absence of the parasite target sequence). Therefore, a high quantity of cells can be applied, as the larger the sample, the higher the probability of detecting parasitic DNA. Consequently, the quantity of initial material should be adapted depending on the aim of the assay.

DNA extraction from macroparasites is generally performed for genotyping or subtyping parasites; therefore, the performance of the extraction process is not critical [4, 22].

Research of PCR inhibitors is necessary for assessing the quality of molecular diagnosis of infectious diseases. Using NucliSENS EasyMAG, we found a low frequency of inhibitors in DNA from stool samples: this may be associated with the process itself and/or the quantity of stool samples used for extraction (approximately 40 mg) as other commercial kits recommend 200 mg of starting material (QIAamp Stool kit, Qiagen). For plasma and BAL samples, potential PCR inhibition of the M13 plasmid was always found negative and testing a dilution of the positive DNA sample via the *Aspergillus* PCR showed a partial inhibition in most cases which would result in an underestimation of the fungal load. Although the specific PCR seems to be the optimal test to evaluate inhibition, it is only applicable to the positive samples, so use of an external system is necessary even if its sensitivity to inhibitors is different.

The current easyMAG protocols do not support direct extraction from blood volumes exceeding 250 μL. If larger volumes are used, residual hemoglobin inhibits subsequent PCRs. Re-extraction protocols may resolve this problem without resulting in a loss of material, even using the silica matrix utilized for the initial extraction.

Our results demonstrate that affinity of the silica matrix for the DNA varies with the conformation of the DNA and that the amplitude of the difference depends on the DNA concentration. This could partially explain the increased quantity observed for kinetoplastic DNA PCR with a low density of parasites and a medium density of host cells. Furthermore, the NucliSENS easyMAG system allows for simultaneous extraction of both DNA and RNA. As these nucleic acids are not dissociable, extraction experiments, especially those assessing the saturation of magnetic silica, should take into account the fact that the silica matrix also adsorbs RNA and thereby affects the extraction yield of DNA. Stability of the lysates constitutes a supplementary advantage, as the extraction process can be delayed without loss of DNA and Boom lysis buffer blocks parasite development (e.g., stages and multiplication) immediately after sampling.

## 5. Conclusion

Nucleic acid extraction largely influences the quality and accuracy of the molecular diagnosis of parasitic diseases. Despite considerable progress regarding innocuity, convenience, and sample process automation, each user must test and consider the limits of each methodology. The advantage conferred by the NucliSENS easyMAG system relies on the high dissociation activity of lysis buffer, rendering PK digestion dispensable in most cases. However, preliminary mechanical disruption is necessary to yield sufficient levels of nucleic acids extracted from fungi and cysts. This system enables the quantitative extraction of DNA from paucicellular samples up to 10^5^ cells, thereby allowing for a complete extraction process in less than 1 h.
